# A pathway-based data integration framework for prediction of disease progression

**DOI:** 10.1093/bioinformatics/btt610

**Published:** 2013-10-24

**Authors:** José A. Seoane, Ian N. M. Day, Tom R. Gaunt, Colin Campbell

**Affiliations:** ^1^MRC Centre for Causal Analyses in Translational Epidemiology, ^2^MRC Integrative Epidemiology Unit, School of Social and Community Medicine, University of Bristol, Clifton BS8 2BN, UK and ^3^Intelligent Systems Laboratory, University of Bristol, Bristol BS8 1UB, UK

## Abstract

**Motivation:** Within medical research there is an increasing trend toward deriving multiple types of data from the same individual. The most effective prognostic prediction methods should use all available data, as this maximizes the amount of information used. In this article, we consider a variety of learning strategies to boost prediction performance based on the use of all available data.

**Implementation:** We consider data integration via the use of *multiple kernel learning* supervised learning methods. We propose a scheme in which feature selection by statistical score is performed separately per data type and by pathway membership. We further consider the introduction of a confidence measure for the class assignment, both to remove some ambiguously labeled datapoints from the training data and to implement a *cautious classifier* that only makes predictions when the associated confidence is high.

**Results:** We use the METABRIC dataset for breast cancer, with prediction of survival at 2000 days from diagnosis. Predictive accuracy is improved by using kernels that exclusively use those genes, as features, which are known members of particular pathways. We show that yet further improvements can be made by using a range of additional kernels based on clinical covariates such as Estrogen Receptor (ER) status. Using this range of measures to improve prediction performance, we show that the test accuracy on new instances is nearly 80%, though predictions are only made on 69.2% of the patient cohort.

**Availability:** https://github.com/jseoane/FSMKL

**Contact:** J.Seoane@bristol.ac.uk

**Supplementary information:** Supplementary data are available at *Bioinformatics* online.

## 1 INTRODUCTION

Within the biomedical sciences it is increasingly common to derive multiple types of data from the same individual. A good example is the Cancer Genome Atlas (cancergenome.nih.gov) in which gene expression (EXP) array, microRNA array, methylation and copy number variation (CNV) data are derived from the majority of tumor samples. By using multiple types of data derived from a given sample, we can understand linkages between attributes within each type of data. Also, by maximizing the information content, models that use all the available data are intrinsically more powerful than models that use only one data type. For these reasons there has been an increasing interest in data integration methods, both for unsupervised (Agius *et al.,* 2009; Huopaniemi *et al.,* 2010; Rogers *et al.,* 2010; Savage *et al.,* 2010; Yuan *et al.,* 2011) and supervised learning (Bach *et al.,* 2004; Gönen and Alpaydin, 2011; Lanckriet *et al.,* 2004; Rakotomamonjy *et al.,* 2008), and their use with genomic datasets.

**Fig. 1.**
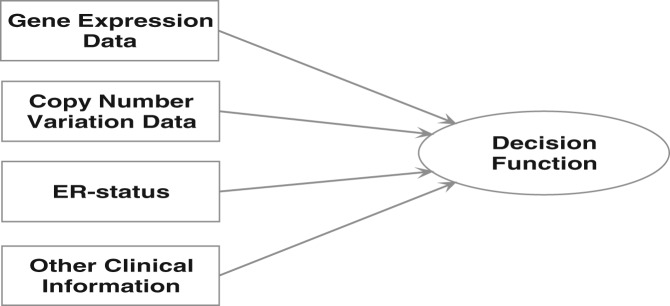
With multiple kernel learning, different types of data are encoded into data objects called *base kernels*. For the METABRIC breast cancer dataset, EXP, CNV, ER status and clinical data are handled by separate base kernels

For supervised learning with multiple types of input data, the decision function will need to successfully integrate the different components of the input data. One way of doing so is to create a committee of decision functions, each handling a separate component of the data, and feed these decisions into an integrative decision function for the final outcome decision. Of course, different types of data will have different degrees of informativeness and consequently we need to be able to weight the contribution of different members of the committee accordingly. One way of doing so is to associate a confidence measure with the vote of individual committee members and use these probabilistic measures to define their relative contribution to the final decision. In this article, though, we follow the more direct route of encoding each type of data into objects called *kernels* and using a weighted combination of these in the final decision function, an approach called *multiple kernel learning* (MKL) (see Fig. 1). Kernels encode the similarity between data objects (Shawe-Taylor and Cristianini, 2004; Campbell and Ying, 2011). In this article, learning is performed using *composite kernels*, which are a linear combination of a large set of *base kernels*, encoding particular types of data.

In Section 2.2, we also consider probabilistic MKL. By restricting prediction to high confidence instances only we can further improve predictive accuracy. In Section 3.2, we apply these methods to the METABRIC data for breast cancer (Curtis *et al.,* 2012) with an application to prediction of mortality risk. Of course, there have been a number of other studies predicting breast cancer outcome using clinical data (e.g. Wishart *et al.,* 2010) or EXP data (e.g. Buyse *et al.,* 2006) alone but few that combine and weight the significance of these different prognostic indicators.

There are alternative methods for supervised learning using multiple types of data, and we will pursue a comparison of our method against these alternatives in Section 3.2.3. We consider methods proposed by Chen and Zhang (2013), Chen *et al.* (2009), Lê Cao *et al.* (2010) and Witten and Tibshirani (2009). Furthermore, in a recent competition by Sage Bionetworks (the DREAM Breast Cancer Prognosis challenge), EXP, CNV and clinical data from the METABRIC dataset were made available for evaluating the performance of different approaches for predicting breast cancer survival. The model that gave the best results (Chen *et al.,* 2013) was an ensemble of different methods, including Cox regression based on the Akaike Information Criterion, a Generalized Boosting Model and *k*-nearest neighbors. This model included prior knowledge based on the selection of groups of genes. In Bilal *et al.* (2013) the authors analyzed several models submitted to this competition, including Random Forest, Lasso-based regression models, Elastic Nets and boosting and ensemble models, and thus we compare with these.

### 1.1 Multiple kernel learning

**Fig. 2.**
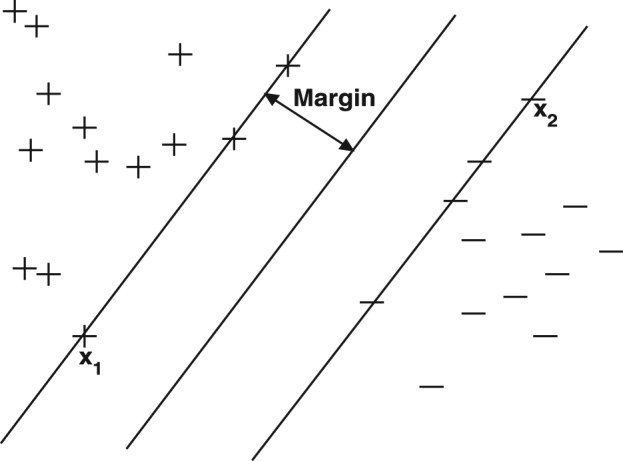
The argument inside the decision function of a classifier is 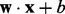. The separating hyperplane corresponding to 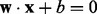 is shown as a line in this 2D plot. This hyperplane separates the two classes of data with points on one side labeled 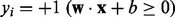 and points on the other side labeled 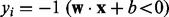

Kernel-based learning machines (Scholkopf and Smola, 2002; Shawe-Taylor and Cristianini, 2004), such as Support Vector machines (SVMs), are a well-studied class of methods for classification problems. For binary classification with two well-separated classes of data (Fig. 2), the learning task amounts to finding a *directed hyperplane*, that is, an oriented hyperplane such that datapoints on one side will be labeled 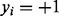 and those on the other side as 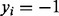. The directed hyperplane found by an SVM is intuitive. It is that hyperplane, which is maximally distant from the two classes of labeled points located on each side. The closest such points on both sides have most influence on the position of this separating hyperplane and are the *support vectors*. The distance between these support vectors and the separating hyperplane is the *margin*. The separating hyperplane is given as 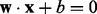 where *b* is the *bias* and **w**, the *weights* ( denotes the scalar product). With datapoints 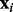 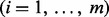 having corresponding labels 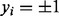, the *decision function* is therefore 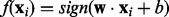. Therefore, datapoints are correctly classified if 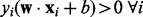. The decision function 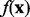 is invariant under a positive rescaling of the argument inside the *sign*-function. This leads to an ambiguity in defining the margin. Hence we implicitly fix a scale for (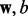) by setting 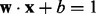 for the closest points on one side and 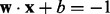 for the closest on the other side. Let 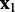 and 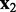 be two support vectors on both sides (Fig. 2). If 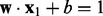 and 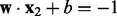, we deduce that 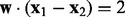. For the separating hyperplane 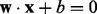, the normal vector is 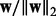 (where 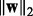 is the square root of 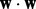). Thus the margin is half the projection of the vector 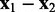 onto the normal vector 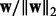 that gives 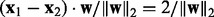. Therefore, the margin is 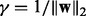. Maximizing the margin is therefore equivalent to minimizing:
(1)
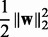

subject to the constraints:
(2)
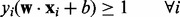

As a constrained optimization problem, the above formulation can be reduced to minimization of a *Lagrange function*, consisting of the sum of the objective function and the *m* constraints multiplied by their respective *Lagrange multipliers*, 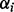 (that satisfy 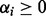). This is the *primal* formulation of an SVM:
(3)
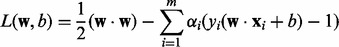



At the minimum, we can take the derivatives of 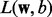 with respect to *b* and **w** and set these to zero. This gives the conditions 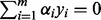 and 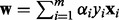. Substituting **w** back into 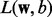 we get the *dual formulation*:
(4)
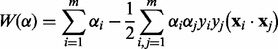

which must be *maximized* with respect to the 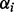 subject to the constraints:
(5)
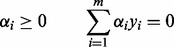



The advantage of the learning task in (4, 5) is that it is constrained quadratic programming from optimization theory, and hence it is a concave problem with a unique solution.

Having found those 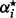, which optimize (4), the predicted label for a new datapoint **z** is given by the sign of
(6)
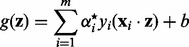

where *b* is the *bias*:
(7)
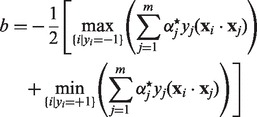



Many datasets are not linearly separable. An appealing property of kernel-based methods, such as SVMs, is that we can map input data into a higher dimensional space, called *feature space*, where the datapoints are linearly separable. With a mapping 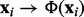 to feature space, from (4) we see that datapoints are represented by a mapped dot product in this higher dimensional space i.e. by 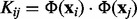. *K_ij_* is called the *kernel matrix*, and we can construct kernels for discrete and continuously valued data and other data objects such as graphs and text strings. A particular choice of kernel amounts to an implicit choice of mapping function though, in practice, we do not need to know the form of this mapping function.

We can therefore construct classifiers with a decision function dependent on a variety of different types of input data. With different types of data encoded in different kernels, this approach is called MKL (reviewed in Campbell and Ying, 2011; Gönen and Alpaydin, 2011). A common approach to MKL is to construct a composite kernel as a linear combination of base kernels:
(8)
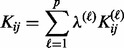

where 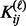 is the base kernel derived from each type of data 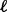 and there are assumed *p* such types of data. The *kernel coefficients*, 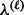, are subject to the constraints:
(9)
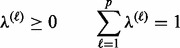

and so the objective function to optimize in 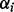 and 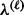 is given by
(10)
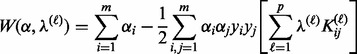

which we optimize via
(11)
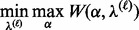

subject to the constraints (5) and (9). This is a linear programming problem in 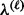 and a quadratic programming problem in 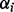 and could thus be approached as a quadratically constrained linear programming problem (Bach *et al.,* 2004), for example.

The kernel coefficients 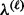 weight the significance of particular kernels and are therefore a measure of the relative importance of different types of data in the final decision function. The different types of data that are input to this decision function will likely have different intrinsic scales. Thus, to account for this variability across datasets, all base kernels are normalized to unit trace norm in the experiments discussed later in the text.

## 2 METHODS

After MKL is complete, the kernel coefficients, 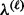, indicate the relevance of different types of data. Thus, if 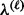 is zero then data type 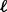 is not relevant or the information it contains may be implicit in another type of data. Thus MKL can indicate that acquisition of certain types of data may not be necessary. For a dataset such as METABRIC (Curtis *et al.,* 2012), the component types of data can have a large number of features and the large majority of these features are likely to be irrelevant to prediction of mortality risk. If irrelevant data substantially outweighs relevant data then we must consider feature selection strategies. In the context of multiple types of data, this feature selection would need to be performed differently per type of data.

### 2.1 Feature selection

In this article, we start by considering feature selection in the context of MKL. We will use a large number of kernels, with variable numbers of features per kernel. Thus the algorithm finds which kernels, and hence which features per data type, are most relevant for the given classification problem. The feature set per kernel can be chosen through statistical scoring (e.g. by ranking those features most statistically aligned with the class labels) or by biological insight (e.g. by selection of a set of genes known to belong to a specific pathway). To implement this approach we would need to select an MKL method that typically gives a sparse combination of kernel coefficients 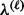. We have selected the SimpleMKL method of Rakotomamonjy *et al.* (2008) because of its observed sparse solution in our previous studies (Damoulas *et al.,* 2008; Ying *et al.,* 2009a, b) and has proven efficiency when the number of kernels is high (Kloft *et al.,* 2011).

SimpleMKL performs an optimization over both the parameters of the SVM (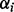) and the kernel coeffients (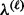) via an iterative gradient descent method. This approach is efficient for high dimensional datasets, as memory consumption remains stable during minimization, in contrast to other implementations based on quadratically constrained quadratic programming (Bach *et al.,* 2004) or semi-infinite linear programming (Lanckriet *et al.,* 2004). Importantly, this particular MKL implementation uses a two-norm regularization leading to a sparse solution in the kernel coefficients.

During construction of the base kernels, 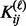, features were grouped into sets. The features in a specific set can be grouped by statistical significance. We used the MATLAB bioinformatics toolbox *rankfeature* function for this purpose. When grouping features by statistical score, we found best results could be achieved using the *t*-test measure, using the class labels of the training set. Once the features in each set are ranked by statistical significance, an individual base kernel was constructed for each set of the first 2, 3 and up to *N* features.

To give further flexibility in terms of the kernel function, for each individual set of features, we used several different types of kernel matrix. We used a linear kernel, as some of the data types had many features (e.g. the EXP and copy number variability data) and so we are considering a sparse set of datapoints in a high dimensional space. Thus it is reasonable to assume datapoints from each class belong to linearly separable sets and therefore a linear kernel is sufficient. We further used polynomial base kernels with 2 and 3 degrees of freedom and non-linear Gaussian kernels. Given our remarks about the separability of the data, we found the method gave a value of zero for the kernel coefficients for the Gaussian kernels. Thus the decision functions were only dependent on linear and polynomial kernels in our experiments in Section 3.2.

As a means for incorporating further biological information, we derived additional base kernels each with a feature set based solely on genes known to be members of a specific pathway. The pathway information was derived from KEGG (Kyoto Encyclopedia of Genes and Genomes. http://www.genome.jp/kegg/). We discuss these in more detail in Section 3.3.

The algorithm therefore has significant flexibility over the set of base kernels used in constructing the most appropriate decision function. Once the algorithm reached an optimum for the objective function, the large majority of the kernel coefficients 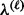 had a value of 0.0 in subsequent experiments and thus the corresponding kernels do not contribute to the decision function. Non-zero coefficients indicate the informative kernels. In the experimental Section 3.2, we consider the performance of the classifier based solely on single data types and multiple data types in addition to performance with and without feature selection.

### 2.2 Introduction of a confidence measure

For many medical prediction problems, it would be useful to have a confidence measure associated with a predicted label. For classification problems using MKL, several dedicated schemes have been proposed that associate a probabilistic confidence measure with the class label. Damoulas *et al.* (2008) proposed two schemes based on variants of the Relevance Vector Machine, and Gönen (2012) proposed a variational Bayes approach. The construction of Gaussian process models that use multiple types of input data has also been considered (Archambeau and Bach, 2011). Some of these schemes have had only limited success. Thus, in Damoulas *et al.* (2008), we found that the use of probabilistic assumptions led to a test accuracy less than that achievable by non-probabilistic classifiers.

Given the limited performance of some proposed probabilistic MKL schemes, we decided to use a simple extension of current non-probabilistic methods, to introduce a confidence measure. Specifically, most MKL methods have an intrinsic measure of confidence. Thus in (6) we introduce the margin distance 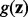: the larger the absolute value of 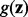 the greater the degree of confidence in the predicted label. To interpret 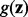 as a probability measure, we fit a posterior probability measure. For binary classification, we use the sigmoid 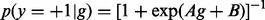. With binary labels 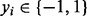, we define 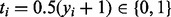. The parameters *A* and *B* are then found by minimizing the negative log likelihood of the training data via the cross entropy error function:

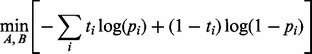

where *p_i_* is the sigmoid probability function evaluated at 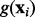 (Platt, 1999). To minimize this function, we used the Levenberg–Marquardt algorithm.

In our experiments in Section 3.2, we used this probability measure in two ways. First, as commented, we investigate whether a gain in test accuracy can be achieved by restricting prediction to a smaller cohort of patients for which high confidence predictions can be made, declining prediction on the remainder. Second, these datasets have input noise due to variability in experimental measurements and the heterogeneity within tumor samples. In addition, there is label noise, as patients first present at various stages of disease progression. Given this consideration, we also used the probabilistic measure on the training examples to remove training examples with ambiguous labels. Thus, for example, in an experiment outlined later in the text, we remove all training examples with an associated probability measure for the label below 0.8.

## 3 EXPERIMENTAL RESULTS

### 3.1 The dataset

In this study, we consider prediction of mortality risk using breast cancer data from the METABRIC project. The METABRIC data consists of clinical data, such as survival period and data derived from EXP and CNV. This dataset is derived from a collection of 2000 clinically annotated primary breast cancer specimens with EXP and CNV data derived from each sample, as described in Curtis *et al.* (2012). The expression data have 48 803 probes or features (based on an Illumina HT 12v3 platform) with data normalized as described in Curtis *et al.,* 2012. The copy number data were extracted using the Affymetrix SNP 6.0 platform, normalized and matched in 19 607 gene regions (Curtis *et al.,* 2012). Some further clinical measurements were available in addition to clinical outcomes. In our study, we have used the following: the disease and treatment group [(i) lymph node negative without chemotherapy, (ii) Estrogen Receptor (ER) positive, lymph node positive, no chemotherapy but hormone therapy, (iii) ER negative, lymph node positive and chemotherapy and (iv) others], grade of disease, stage, histological type (Invasive Ductal Carcinoma IDC, Invasive Lobular Carcinoma ILC, IDC+ILC, IDC tubular, IDC mucinous, other, other invasive and benign), ErbB2 Receptor (HER2) status, age, tumor size, Nottingham Prognostic Index, tumor cellularity and PAM50 (PAM50 Breast Cancer Intrinsic Classifier) subtype by expression clustering. As outcome variable we considered a simplified survival analysis, consisting of prediction of survival versus non-survival at 2000 days. In subsequent experiments, we used a dataset of 387 survival cases and 252 non-survivors at 2000 days (a subset of the METABRIC data, as some patients were followed-up for < 2000 days with no record of mortality and would not qualify, for example).

### 3.2 The results

#### 3.2.1 Using EXP and CNV data only

**Fig. 3.**
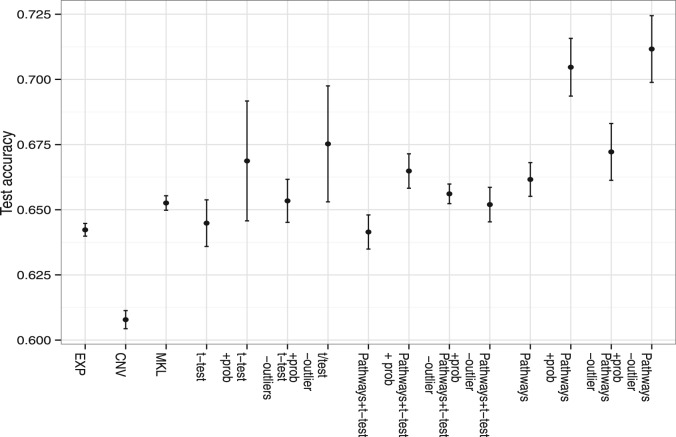
This figure shows results for experimentation with EXP and CNV data. The *y*-axis is the test accuracy expressed as a fraction and the *x*-axis indicates the experiment considered. The figure compares the results of using an SVM on each of the two datasets separately (*EXP* and *CNV*), multiple kernel learning (*MKL*), feature selection by statistical score (*t-*test, in combination with other measures), the use of pathway-based kernels (*Pathways*) and the use of a probabilistic score associated with the classifier, to remove ambiguously labeled training points (*−out*) and/or restrict prediction to high confidence (*+prob*), as discussed in the text

In our first round of experiments, we therefore considered the set of extensions of MKL learning outlined in Sections 2.1 and 2.2, using EXP and CNV data only. With reference to Figure 3, we first estimated the test accuracy using the expression array data only with no feature selection (EXP). Next we performed the same experiment on the CNV data, again with no feature selection. In this case, we use a standard SVM for training and test purposes. With this dataset, we determined the error bars in Figure 3 using 5-fold cross-validation with a prior random reshuffling of the sample order. With EXP we get a test accuracy of 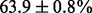 and for CNV 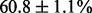. If we perform MKL with these two datasets, we improve the test accuracy (getting 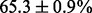) because we are using more information. Next we enabled feature selection with MKL (labeled t-test in Fig. 3). On the training data only, we used the t-test to rank features in both datasets according to alignment with the class labels. We constructed kernels using the top 2 through to the top 15 features for both datasets. There was no observed improvement in performance with this strategy, with a test accuracy of 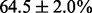. Owing to computational cost, we did not enlarge the set of base kernels beyond the top 15 features per dataset.

**Fig. 4.**
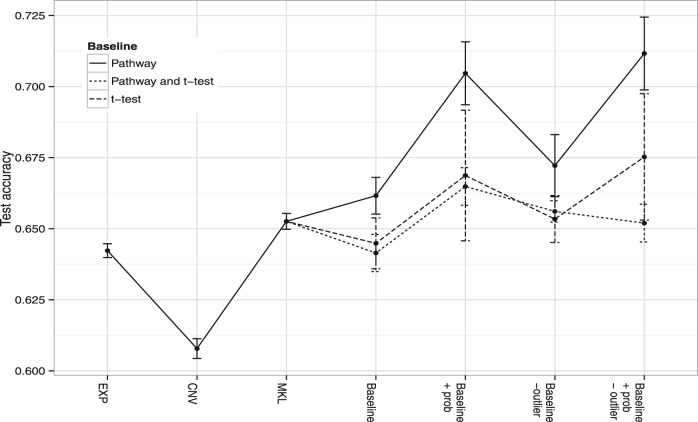
In this figure, we compare the use of pathway-based kernels only (*Pathway*) with addition of kernels based on a statistical scoring on the training data (*Pathway and t-test*) and the use of purely statistical kernels (*t-test*). The *y*-axis gives the test accuracy as a fraction. The use of kernels based on statistical ranking (*t-test*) seems to degrade test accuracy, and it is best to use kernels based solely on pathway information

Next we introduced a set of base kernels each of which exclusively used those genes belonging to known pathways from the KEGG database (these are marked *Pathways* in the figure). Feature selection by statistical score alone did not offer improved performance consistently, nor did using these kernels together with pathway kernels. However, feature selection by pathway membership alone appeared to give a consistent improvement with the added advantage of biological interpretability: we illustrate this gain in Figure 4. Our next variation was to introduce the probabilistic measure on the output labels. In this case, we started by only giving predictions with the most confident cases, that is, the *P*-value must be ≥0.95 (probabilistic measure, PROB in figures). This did improve test accuracy to 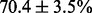, the large spread being because of the smaller size of the predicted set. Though some gain in test accuracy is achieved, it was at the cost of loss of prediction on part of the patient cohort. Removal of training examples with posterior probabilities <0.8 (*OUTLIER* in the figures) gave 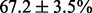, while the combination of the two strategies gave 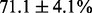.

#### 3.2.2 Using additional kernels based on clinical information

Breast cancer is known to have clinically defined subtypes. One broad distinction is between estrogen-receptor positive (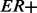) and negative (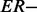) cases. The prognosis for 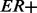 disease is much better than 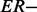 disease and within the category of 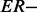 disease there is further differentiation between 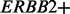 (or 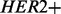) and the triple-negative subtype (that has 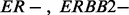). Again the clinical outcomes for these latter subtypes are distinct. Consequently, incorporation of such clinical information is likely to boost test accuracy. In this section, we consider the additional incorporation of *ER*-status information alone and then the use of all the clinical information mentioned in Section 3.1, encoded into additional base kernels. In certain cases, such as *ER* status, the information is binary valued. In this case, we used Boolean variables to encode the kernel. If this additional clinical data contained >2 label values, then we used a set of Boolean variables to encode the label class and hence construct the kernel.

**Fig. 5.**
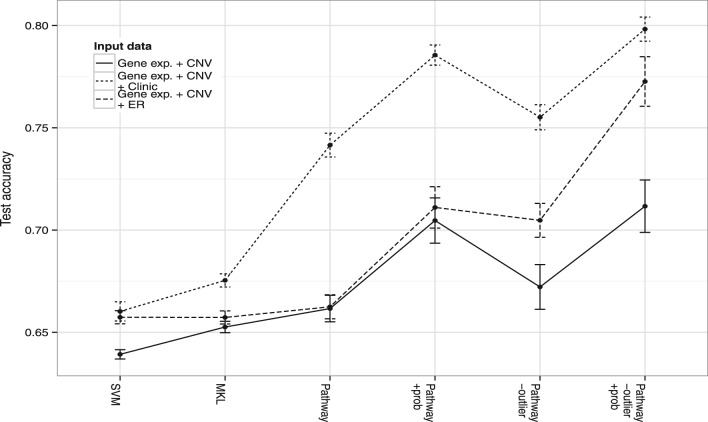
This figure shows the results of a comparison between test accuracies with three different combinations: EXP array and CNV data only (EXP+CNV), the latter kernels supplemented by kernels based on *ER*-status (*EXP+CNV+ER*) and the latter kernels supplemented with kernels for all the clinical covariate data (*EXP+CNV+clinic*). The clinical data includes *ER*-status. The *y*-axis gives the test set accuracy for survival to 2000 days. The best combination is to use all the clinical data, EXP and CNV data encoded into pathway-based kernels, together with removal of ambiguously labeled training datapoints *−outlier* and the use of a cautious classifier *+prob* via a probabilistic confidence measure

From Figure 5 we see that the incorporation of further clinical information significantly improves performance. Using EXP array and CNV data only (*EXP+CNV*) gave the weakest test performance even if we used MKL and pathway-based kernels. A significant gain was made if we supplemented these kernels with kernels based on *ER*-status (*EXP+CNV+ER*). The best performance was achieved if we supplemented the latter kernels with kernels for all clinical covariate data (*EXP+CNV+clinic*). This included *ER*-status and excluded the survival indicator, of course. Using pathway-based kernels, removal of ambiguous training examples and using a cautious classifier, we get a test accuracy of 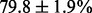. Using normalized kernel coefficients, we found that the highest weight kernel was a pathway kernel associated with immune response (KEGG:hsa04672). The second highest weighted kernel was tumor size and clinical data are generally highly ranked. The ninth most informative was the Nottingham Prognostic index, a combined measure of tumor size, lymph node involvement and grade. Age was 11th rank, histological type 14th, tumor group 34th and PAM50 was 40th.

**Fig. 6.**
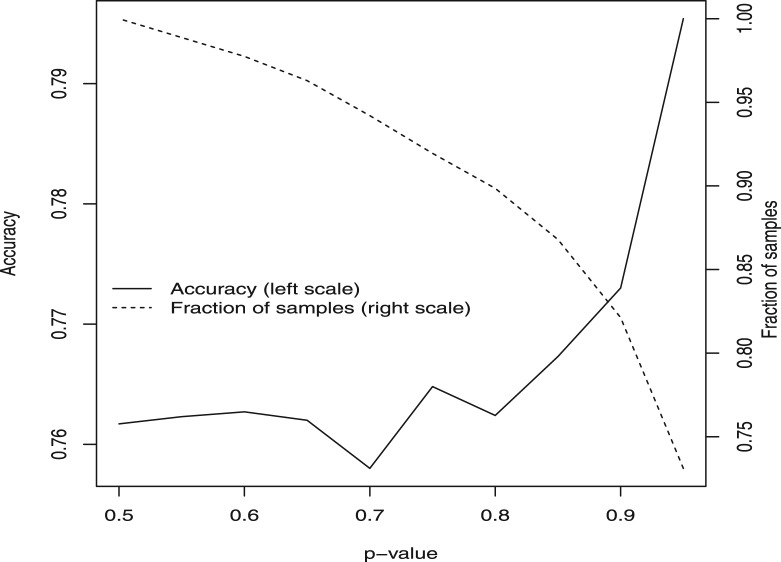
As the cut-off *P*-value is changed (*x*-axis), determining the lowest tolerated confidence level, the prediction accuracy alters: this is given by the left-hand axis and the solid curve. As the *P*-value is increased the accuracy can increase but the fraction of the data on which predictions are made decreases (dashed curve), reducing to 69.2% of the patient cohort at a cut-off of *P* = 0.95

In Figure 5, the best overall test accuracy was achieved using all available data from EXP array and copy number variability through to clinical measures and *ER*-status. MKL was used to learn and weight the significance of these different types of data. Furthermore, pathway information was used implicitly by using pathway data-based kernels for both the EXP array and the CNV array data: that is, the respective kernels are constructed using only those genes or CNV regions with known membership of a given pathway. To improve accuracy, we further used a probabilistic measure associated with the class label. Firstly, we used the training set to construct this label and then removed those training points with an ambiguous label (in this case the probability of membership of the principal class was <0.8). With the predictions, we used this probabilistic measure to remove predictions with an associated ambiguity for the class assignment (the probability of membership of the given class was <0.95). Consequently, the predictor only made predictions on a cohort of 430 samples from 621 with a cut-off on the probability of *P* = 0.95. In Figure 6, we illustrate variation in test accuracy and the fraction of patients for which predictions are made, when using a cut-off on the confidence measure and removing ambiguously labeled datapoints.

#### 3.2.3 Comparison with other approaches

**Fig. 7.**
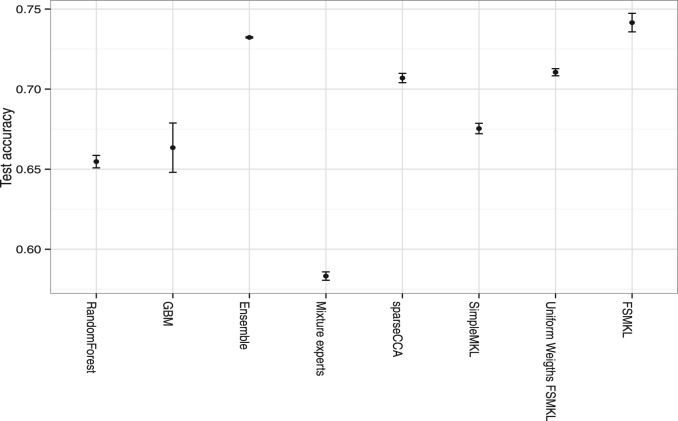
This figure gives the test accuracies for the method outlined in this article (*FSMKL*), against other data integration techniques that are described in the accompanying text (5-fold cross validation was used). All comparisons are with (*EXP+CNV+clinical*) data, except the mixture of experts model, which only uses (*EXP+clinical*)

To validate the performance of our approach, we compared it with previous data integration methods, which have been suggested as state-of-the-art. With a variety of prospective methods, we selected representative algorithms from several different approaches. In the case of ensemble models, we chose a bagging method that has been widely used in genomic analysis, based on Random Forest (Breiman, 2001) and using the R package randomForest (RandomForest in Fig. 7). For a boosting model, we chose a Generalized Boost Regression Model (GBM in the figure), an extension of Friedman’s Gradient Boosting Machine (Friedman, 2001), which is implemented in the R package gbm. As a further comparator, we chose an ensemble implementation based on blending classification and regression models, via a greedy stepwise approach, and proposed by Caruana *et al.* (2004), available in GitHub (https://github.com/zachmayer/caretEnsemble) and modified to work with multiple data sources (Ensemble in the figure). This particular model emulated the winning ensemble model used in the Breast Cancer Prognosis Challenge (Chen *et al.*, 2013), which combined a boosting regression model (GBM), a regularization model (Elastic-Net Generalized Linear Model, using the R package glmnet) and k-nearest neighbors (using the R-package Caret implementation). We further included a mixture of experts model (Lê Cao *et al.*, 2010, mixture experts in the Figure). This latter implementation only permits integration of two datasets: we chose EXP and clinical data, for best competitive performance. We also compared our model with the supervised sparse Canonical Correlation Analysis (CCA) implementation described in Witten and Tibshirani (2009) (sparseCCA). Finally, to compare with a baseline MKL without optimization of the kernel coefficients, we included MKL with uniform kernel weights (Uniform Weights FSMKL). The approach outlined in this article is labeled FSMKL (for MKL learning with feature selection), and is given as 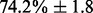, without the improvement derived from cautious classification, for comparison (see Fig. 6).

We compared our method with these alternatives using the same sets of data, the exception being mixture of experts where only EXP and clinical data were used. For each method, we ran tests on validation data, if parameter values had to be set, and all methods were evaluated using 5-fold cross-validation. As illustrated in Figure 7, the MKL approach proposed in this article outperformed the test accuracies for the stated alternative methods. Surprisingly, the third best result was obtained by using the same MKL implementation, but with uniform kernel coefficients. This suggests that the relative weighting of the different types of data is less important than including a wide variety of different types of data, including pathway information. Although some of these alternative methods allow for gene prioritization, or can indicate which type of data has most influence on sound prediction, our approach permits identification of the most important pathways, and the most important genes in each pathway, while also obtaining a higher test accuracy compared with these alternative approaches.

### 3.3 Interpretation of the pathway-based kernels

Because we are using pathway-based kernels, the relative value of their respective kernel coefficients, 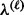, will indicate their relative influence on survival. In all, up to 146 pathway-based kernels were used by the various methods considered in this article (i.e. they had 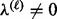). Of these, 98 have been cited in the literature as having an influence on survival outcome for at least one type of cancer, with the majority related to survival outcome in breast cancer. The Table in the associated Supplementary Material lists all these pathways together with associated *PUBMED* links. In this section, we therefore discuss these pathways and similarities between our analysis and the cluster model and pathways discussed by Curtis *et al.* (2012).

Using unsupervised learning, Curtis *et al.* identified 10 putative clusters or disease subtypes and discussed pathway enrichment within these clusters. The two clusters with worst survival outcome were labeled *Int2* and *Int5*. For their pathway analysis, Curtis *et al.* used the Ingenuity pathway analysis software, which includes other pathways in addition to KEGG pathways. For this reason, a direct comparison is not possible, but we can consider if pathways present in our analysis are also covered in the pathway analysis of Curtis *et al.* Using all the clinical data, the EXP and CNV data types, and with survival as outcome, our MKL method used 81 pathway-based kernels, of which 27 are enriched in the *Int2* cluster and 21 in the *Int5* cluster. If we restrict to EXP, CNV and ER-status only for input data, our MKL method used 83 pathways, of which 28 are present in the *Int2* cluster and 23 in the *Int5*. In the Supplementary Material, we give a complete list of the pathways used, associated scores and scores of Curtis *et al.* (these scores are not comparable).

With our MKL analysis, one of the highest significance pathways is RNA transport, which has been previously reported as a key pathway in the recurrence of non–small-cell lung cancer (Lu *et al.,* 2012). Other significant pathways were cell adhesion molecules, endocytosis, the insulin signaling pathway and the mTOR signaling pathway. The arachidonic acid metabolism pathway (Iwamoto *et al.,* 2011; Nassar *et al.,* 2007) and N-glycan biosynthesis (Dennis *et al.,* 1999) both feature and have reported associations with breast cancer development, as does SOCS [Suppression of cytokine signalling pathway (Sasi *et al.,* 2010)], which is a negative regulator of the JAK-STAT signalling pathway and it is associated with improved clinical outcome in breast cancer. As also reported by Curtis *et al.,* 2012, the Systemic Lupus Erythematosus pathway featured and, with an association to ER-status, also has an association with survival-status. 

## 4 DISCUSSION

We now discuss some broad conclusions that can be drawn from this study, various ways in which classifier test accuracy can be further improved and other contexts in which we could apply the method outlined.

Two key conclusions coming from our study are the importance of incorporating prior knowledge and performing feature selection. Prior knowledge was represented by pathway information. Where appropriate, data were grouped into clusters representing their particular pathway membership. Using feature selection within each such cluster, we use the most representative features within each pathway. Because of the sparse nature of this particular MKL implementation, we can select a set of pathways that are most relevant to survival prediction.

As expected, classifiers that can use all the available data are more powerful that those that use only one type of data. MKL methods also have the advantage that they weight the contribution of individual data types, and thus indicate their relative significance. Our study highlighted the importance of using all available clinical data alongside expression array and CNV data. In addition, expression and CNV data were best incorporated into the classifier by using pathway-based kernels. Further improvements came from using a *cautious classifier* that only makes predictions on a restricted class of high confidence cases and by removal of ambiguously labeled samples from the training data. These last improvements highlight the importance of using a confidence measure associated with the label assignment and motivate further work on devising robust probabilistic classifiers for use with MKL.

Using these various measures, predictive performance moved from ∼64% for prediction with EXP array data alone, to almost 80%, with the qualification that prediction is made on 69.2% of individuals. However, it is reasonable to expect that this test accuracy can be improved beyond 80% through the use of additional types of data and further refinement of the method. MicroRNA array data, methylation data and condensed information from images of tumor biopsies are complimentary types of data, which could provide additional base kernels, in addition to string kernels (Campbell and Ying, 2011; Shawe-Taylor and Cristianini, 2004), incorporating sequence data. Furthermore, expression by certain individual genes [e.g. *p27* (Alkarain *et al.,* 2004)] or small sets of genes [e.g. associated with *TP53*, (Jamshidi *et al.,* 2013)] has documented correlation with survival status, and these genes could be given extra weight by assigning them individual base kernels.

For the methodology, there are some further directions that could be considered. Rather than using KEGG pathway data, we could investigate other approaches to feature selection, such as filtering features based on Gene Ontology labels. The kernel coefficients would then indicate which Gene Ontology labels are most relevant to predicting survival outcome. If one of the two classes is viewed as more clinically important than the other, then we could use an asymmetric soft margin (Veropoulos *et al.,* 1999) during SVM training: this improves test accuracy on one class, at the expense of accuracy on the other. The SimpleMKL method used in this article has associated publically available software (http://asi.insa-rouen.fr/enseignants/arakoto/code/mklindex.html); it is an effective and representative MKL algorithm and it gives a sparse representation over the set of base kernels. However, a large number of MKL methods have been proposed (Gönen and Alpaydin, 2011) and some methods with a less sparse solution may give higher accuracy (Kloft *et al.,* 2011). In short, additional data, further refinement of the method and the use of a cautious classifier could lead to a test performance nearer 90%. This performance, though, would be achieved at the cost of a wide range of genomic and clinical measurements and does not result in prediction with all patients.

Nomograms and simple clinical measures such as ER-status are reliable indicators of disease progression for breast cancer. A predictive method, such as that described, would need to be competitive against these. However, it is in other contexts that similar studies could be effective. Thus, for prostate cancer, there is a well-recognized problem distinguishing aggressive from low-risk cancer. In the US, ∼20% of men will be diagnosed with prostate cancer, whereas only 3% would die from the disease (Altekruse *et al.,* 2010). With limited ability to predict risk, many tumors are unnecessarily labeled as high risk and treated aggressively. It would be interesting to see if the test accuracies stated in this article can be achieved with prostate cancer and other cancers. This would require similar large datasets with a broad range of genomic and clinical measurements. To get good predictive performance, the dataset would need to contain a sufficient number of aggressive disease examples and not just represent the spectrum of disease observed in the general population—which is numerically weighted toward low-risk disease.

## Supplementary Material

Supplementary Data
