# Supplementary material for: A pathway-based data integration framework for prediction of disease progression
Source: Bioinformatics. 2013 Oct 24;30(6):838–45. doi: 10.1093/bioinformatics/btt610 (PMC3957070; doi:10.1093/bioinformatics/btt610)
Supplement: Supplementary Data [file supp_30_6_838__index.html]

A pathway-based data integration framework for prediction of disease progression — A pathway-based data integration framework for prediction of disease progression — A pathway-based data integration framework for prediction of disease progression — Supplementary Data 

# A pathway-based data integration framework for prediction of disease progression

## Supplementary Data

files

**Files in this Data Supplement:**

- Supplementary Data - pdf file
