# Supplementary material for: A pathway-based data integration framework for prediction of disease progression
Source: Bioinformatics. 2013 Oct 24;30(6):838–45. doi: 10.1093/bioinformatics/btt610 (PMC3957070; doi:10.1093/bioinformatics/btt610)
Supplement: Supplementary Data [file supp_btt610_Supplementary_Material.pdf]

Supplementary Material for 'A pathway-based data integration framework for prediction of disease progression'

José A. Seoane[1], Ian N.M. Day[1], Tom R. Gaunt[1] and Colin Campbell[2]

1. MRC Centre for Causal Analyses in Translational Epidemiology, School of Social and Community Medicine, Oakfield House, University of Bristol, Clifton BS8 2BN, United Kingdom
2. Intelligent Systems Laboratory, Merchant Venturer's Building, University of Bristol, Bristol BS8 1UB, United Kingdom

1. The Data. In this study we used the METABRIC dataset for breast cancer. This data can be obtained from the European Genome-Phenome Archive (EGA) at: https://www.ebi.ac.uk/ega/, reference EGAC01000000010. Access to the dataset must be approved by the specified Data Access Committee.

2. The Software. In this study we used *multiple kernel learning* (MKL), which we conceptually illustrate below:

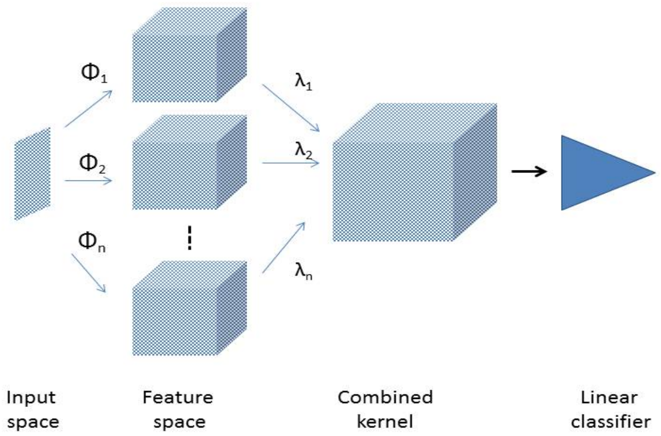

Figure 1: With multiple kernel learning, n different types of data, are encoded into data objects called *base kernels*. These kernel matrices quantify the similarity of data objects in their respective *feature spaces*. Data is mapped to feature space via an implicit mapping function Φ_ℓ defined by the choice of kernel. We derive a *composite kernel* $K_{ij} = \sum_{\ell=1}^{n} \lambda_\ell K_{ij}^{\ell}$ as a linear combination of base kernels. The λ_ℓ quantify the relative significance of different types of data.

We used the SimpleMKL method described in:

- Rakotomamonjy, A. *et al* (2008). SimpleMKL, *Journal of Machine Learning Research*, **9**, 2491-2521.

The MATLAB code for SimpleMKL is available from:

- http://asi.insa-rouen.fr/enseignants/ arakoto/code/mklindex.html

Further MATLAB code used in the project (e.g. for training the probabilistic classifier) can be obtained from the first author, Jose Seoane (*J.Seoane@bristol.ac.uk*).

3. The Pathway-based Kernels. In Section 3.3 of the paper we discuss the pathway-based kernels used in this study. The kernel coefficients λ_ℓ found by the MKL algorithm indicate the relative significance of data ℓ. In the Table below (Table 1) we list Pathway names and the values of λ_ℓ for three studies (using a probabilistic measure and cutoff $p = 0.8$ to remove ambiguously labelled datapoints from the training data and a cutoff of $p = 0.95$ for prediction). Kernels are normalised to unit trace norm and the λ_ℓ sum to unity overall across all the pathway-based and other kernels.
The kernels used differ according to the data available, which includes expression and copy number variation (EXP+CNV), this data supplemented by *ER*-status and this data supplemented by other clinical measures (Clinical). In Section 3.3 we also consider links with the original report for the METABRIC study:

- Curtis, C. *et al* (2012). The genomic and transcriptomic architecture of 2,000 breast tumours reveals novel subgroups, *Nature* **486**, 346-52.

making reference to their *Int2* and *Int5* putative disease subtypes. In two further columns we list pathways which were found enriched in these subtypes (Curtis *et al*). The scores listed in these two columns are not comparable to the λ_ℓ and the Curtis *et al* study is for unsupervised learning whereas our study is for supervised inference. In the final column we list PUBMED IDs for links to previous studies of these pathways, some of which are discussed in Section 3.3.

**Pathway Name**	λ_ℓ in EXP +CNV	λ_ℓ in EXP +CNV +ER	λ_ℓ in EXP +CNV +Clinical	Curtis *et al* Int2	Curtis *et al* Int5	Pubmed(ID) links
ABC transporters		0.033559	0.000985			23288347
Acute myeloid leukemia		0.001378		0.75	1.19	
Adipocytokine signaling pathway	0.004283	0.001991				23104651
African trypanosomiasis	0.134195	0.060504	0.045783			
Alanine, aspartate and glutamate metabolism	0.078918	0.020121	0.007695	0.585		20831783
Aldosterone-regulated sodium reabsorption	0.004718					
Aminoacyl-tRNA biosynthesis	0.076851	0.052629	0.038715			22952576; 20831783
Amoebiasis		0.012966				
Amyotrophic lateral sclerosis (ALS)	0.069929	0.024328	0.008442	0.279		20043860
Antigen processing and presentation	0.056536	0.013096		0.401		21479927
Arachidonic acid metabolism	0.126992	0.053015	0.110905	0.272	0.476	17721268; 21191116
Asthma	0.013037	0.049661	0.068746			
Autoimmune thyroid disease	0.094583	0.02629		0.444		22015293
B cell receptor signaling pathway	0.019986					21394647
Basal transcription factors	0.026157	0.090227	0.059941			
Base excision repair		0.039524	0.032898			17230526
Bile secretion		0.018935	0.034157	2.3	2.42	
Biosynthesis of unsaturated fatty acids	0.017458					20831783
Biotin metabolism	0.019829	0.023815	0.022696			20043860
Butanoate metabolism			0.006146	0.538	0.585	18254968
Butirosin and neomycin biosynthesis			0.004907			
Carbohydrate digestion and absorption		0.035195				
Cell adhesion molecules (CAMs)		0.037951				22292069; 20043860; 17894856
Cell cycle		0.001391	0.007311	0.601	0.651	23336272; 22759382
Chagas disease (American trypanosomiasis)	7E-006					
Citrate cycle (TCA cycle)	0.114664	0.018513	0.019324		0.267	20831783
Collecting duct acid secretion	0.012042	0.033874	0.029998			
Colorectal cancer	0.007179			3.03	0.907	20043860
Complement and coagulation cascades			0.044082			22015293
Cyanoamino acid metabolism			0.003206			
Cysteine and methionine metabolism	0.088221	0.023762	0.037897	0.234		20831783
Cytokine-cytokine receptor interaction			0.015073			22759382; 22015293
D-Arginine and D-ornithine metabolism		0.008857				
D-Glutamine and D-glutamate metabolism		0.020674	0.011036			20043860
Drug metabolism - cytochrome P450		0.002728				20716162; 20574415; 21961651
Drug metabolism - other enzymes	0.03763					
Endocytosis	0.083081		0.027417			22292069
Ether lipid metabolism	0.010137					20043860
Fat digestion and absorption			0.021575			
Folate biosynthesis	0.008352			0.25	0.755	20043860
Glycerolipid metabolism		0.070103	0.024037	3.52	3.23	
Glycerophospholipid metabolism	0.145968	0.078957	0.109364			20043860
Glycine, serine and threonine metabolism		0.019836				20831783
Glycosaminoglycan biosynthesis - heparan sulfate		0.031008	0.053015			
Glycosaminoglycan degradation	0.052699	0.036355				21191116
Glycosphingolipid biosynthesis - ganglio series	0.012802	0.079857	0.001921			
Glycosphingolipid biosynthesis - globo series		0.040046	0.004108			21191116
Glycosphingolipid biosynthesis - lacto and neolacto series	0.009665	0.011058				21191116
Glycosylphosphatidylinositol(GPI)-anchor biosynthesis	0.166829	0.056413	0.089541			20043860
Glyoxylate and dicarboxylate metabolism		0.033762	0.000237		0.529	20043860
Graft-versus-host disease	4.8E-005	0.003129		0.666	0.392	21479927; 22015293
Hematopoietic cell lineage		0.009949	0.023524			20458058; 22015293
Hepatitis C	0.003029					
Histidine metabolism		0.034648	0.007309	1.01	1.08	20831783
Homologous recombination	0.060545	0.033276	0.095827			23364677; 16540687
Huntington's disease		0.011196	0.027719			20043860
Inositol phosphate metabolism		0.009403		0.299		20831783
Insulin signaling pathway			0.0005			15683819; 22292069; 20043860
Intestinal immune network for IgA production	0.078579	0.097819	0.128644			22015293
Jak-STAT signaling pathway	0.099403	0.035611		0.638	0.696	20433750; 20043860
Leishmaniasis	0.000339	0.009869				22015293
Linoleic acid metabolism	0.005113					20043860
Lipoic acid metabolism	0.011993	0.016531				
Long-term potentiation		0.006651				20043860
Lysine biosynthesis	0.017802	0.002526	0.001534			20831783
Lysine degradation	0.028959	0.005841		1.43	1.29	20043860
Malaria		0.001942				
MAPK signaling pathway		0.0269		2.32	2.54	21258408; 11400118
Maturity onset diabetes of the young		0.034089	0.068252			20043860
Melanogenesis			0.012465			20043860
Metabolic pathways	0.023837	0.028638	0.017372			17894856
Metabolism of xenobiotics by cytochrome P450	0.000102			1.15	2.39	20043860; 21191116
mTOR signaling pathway	0.016621					21191116; 17805960; 20043860
Mucin type O-Glycan biosynthesis		0.002686	0.014917			
N-Glycan biosynthesis	0.111709	0.078746	0.078615			10580127
Natural killer cell mediated cytotoxicity	0.067989	0.002562	0.042774			20043860
Neuroactive ligand-receptor interaction			0.002909			17483504
Neurotrophin signaling pathway			0.022185	1.28	1.37	11359788
Nitrogen metabolism	0.012438	6.7E-005		0.234		20043860
Non-homologous end-joining	0.018252	0.047355				
Nucleotide excision repair			0.01195			20508946; 21751198
Olfactory transduction		0.029575				20043860
One carbon pool by folate	0.115314			0.84	1.46	20043860
Oocyte meiosis			0.000848			22046239
Osteoclast differentiation	0.019686	0.003927				20881962
Other types of O-glycan biosynthesis		0.008705			0.283	

**Pathway Name**	$\lambda^{(\ell)}$ in EXP +CNV	$\lambda^{(\ell)}$ in EXP +CNV +ER	$\lambda^{(\ell)}$ in EXP +CNV +Clinical	Curtis *et al* Int2	Curtis *et al* Int5	Pubmed(ID) links
Pantothenate and CoA biosynthesis	0.003667			0.385	0.408	20043860
Parkinson's disease	0.013173	0.03299	0.001254			21114830
Pathogenic Escherichia coli infection		0.012434				20043860
Pathways in cancer	0.055139					
Pentose and glucuronate interconversions	0.038486					
Pentose phosphate pathway	0.000129	0.032764	0.025848	0.793	0.842	20831783
Phagosome	0.008379	0.001736	0.022724			
Phenylalanine metabolism	0.00131		0.024838	1.82	1.91	
Phenylalanine, tyrosine and tryptophan biosynthesis	0.011205	0.004188				19933158
Porphyrin and chlorophyll metabolism		0.002019				20043860; 22136817
PPAR signaling pathway	0.029537		0.002548	1.13	1.23	20043860
Primary bile acid biosynthesis		0.007003	0.019478	2.3	2.42	20831783
Primary immunodeficiency	0.016648					21479927
Prion diseases		0.096994	0.077731			20043860
Progesterone-mediated oocyte maturation	0.006667		0.006708			22046239
Propanoate metabolism	0.0275					2071289
Proteasome	0.089938	0.030921				20043860
Protein digestion and absorption		0.049936				
Protein processing in endoplasmic reticulum			0.017523			22292069
Purine metabolism		0.018934	0.001077			20831783
Pyrimidine metabolism		0.007896	0.013181	0.261		20831783
Pyruvate metabolism		0.009935	0.011341	1.03	1.12	20831783
Regulation of autophagy			0.004615			23104651
Renin-angiotensin system	0.002528	0.025207	0.021006	0.421	0.312	20043860
Retinol metabolism		0.012074				20043860
Rheumatoid arthritis	0.057367					
Riboflavin metabolism			0.004538	0	0.339	
Ribosome			0.012674			20043860
Ribosome biogenesis in eukaryotes		0.005091				22292069
RIG-I-like receptor signaling pathway	0.023082	0.010856				
RNA degradation	0.010072	0.033119	0.020662			
RNA polymerase	0.020168					22759382
RNA transport	0.133083	0.072775	0.079374			22292069
Selenocompound metabolism			0.010811			
Small cell lung cancer	0.014385		0.013242	0	0.384	20043860
SNARE interactions in vesicular transport		0.080697	0.038928			
Sphingolipid metabolism	0.000315	0.12458	0.086686	0.342		21191116; 18058224
Spliceosome			0.01144			
Staphylococcus aureus infection	4E-006	0.007947				
Steroid biosynthesis		0.038189			0.319	21191116; 21191116
Steroid hormone biosynthesis	0.037371					
Sulfur relay system	0.007859		0.002377			
Synthesis and degradation of ketone bodies		0.041141				20043860
Systemic lupus erythematosus	0.058045	0.046132	0.110265	0.345	0.395	15734144; 22292069
T cell receptor signaling pathway						20433688; 22292069; 18834526
Taste transduction	0.089529	0.08309	0.026378			
Taurine and hypotaurine metabolism	0.022575	0.068735		0.579	1.18	
Terpenoid backbone biosynthesis			0.076947			
TGF-beta signaling pathway	1.5E-005	0.053525		0.417	0.28	22759382
Thiamine metabolism	0.009401		0.058481			
Thyroid cancer	1.8E-005			2.67	1.6	20043860
Tryptophan metabolism	0.023641	0.020078		0.535	0.824	20831783; 21191116
Type II diabetes mellitus			0.006002	1	0.278	22292069; 20043860
Tyrosine metabolism	0.007068			1.94	2.07	
Ubiquinone and other terpenoid-quinone biosynthesis	0.009308					
Valine, leucine and isoleucine degradation		0.025937		0.448	0.491	20831783
Vascular smooth muscle contraction		0.005687				
Vasopressin-regulated water reabsorption			0.011845			
VEGF signaling pathway	0.03944	0.002945				20043860; 11857378
Vitamin digestion and absorption		0.105096	0.094445			

Table 1: This table shows the kernel weigths λ_ℓ for EXP+CNV, EXP+CNV+ER and EXP+CNV+Clinic. The int2 and int5 scores reported in (Curtis *et al*) and Pubmed id references of this pathway.
